# Supplementary material for: Synovial-Fluid miRNA Signature for Diagnosis of Juvenile Idiopathic Arthritis
Source: Cells. 2019 Nov 26;8(12):1521. doi: 10.3390/cells8121521 (PMC6952798; doi:10.3390/cells8121521)
Supplement: Supplementary file 1 [file cells-08-01521-s001.pdf]

Synovial fluid miRNA signature diagnoses juvenile idiopathic arthritis

Nadege NZIZA[1,2]*, Eric JEZIORSKI[3,4]*, Marion DELPONT[5], Mailys CREN[1], Hugues CHEVASSUS[7,8], Aurélia CARBASSE[3], Perrine MAHE[3], Hamouda ABASSI[3] , Pauline JOLY MONRIGAL[5], Eric SCHORDAN[6], Alain MANGE[9], Christian JORGENSEN[1,10], Florence APPARAILLY[1,10]*, Isabelle DUROUX-RICHARD[1]*✉

Additional files

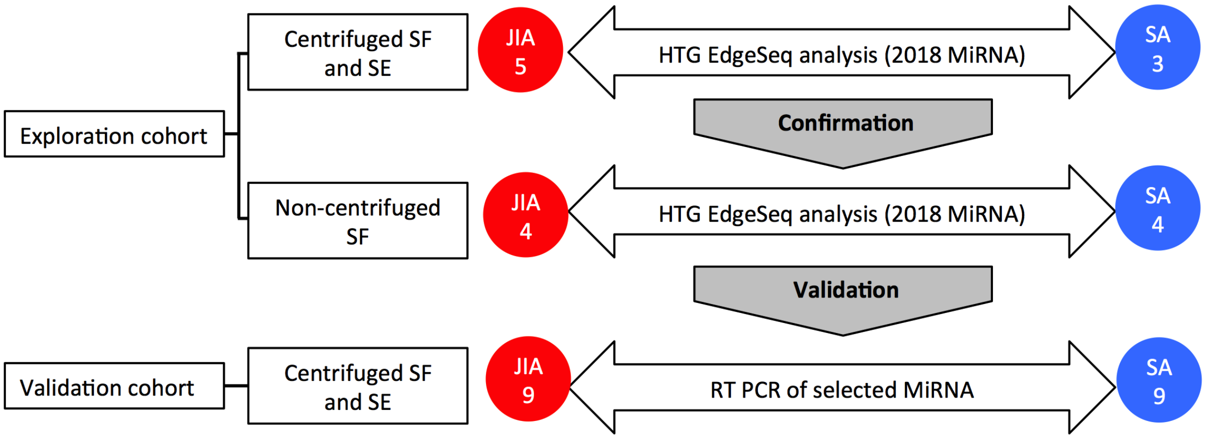

Figure S1. Cohort of synovial fluid and serum samples for miRNA studies. Figure S2. Volcano plot of miRNAs differentially expressed in juvenile rheumatoid arthritis in comparison with septic arthritis identified by WTA.

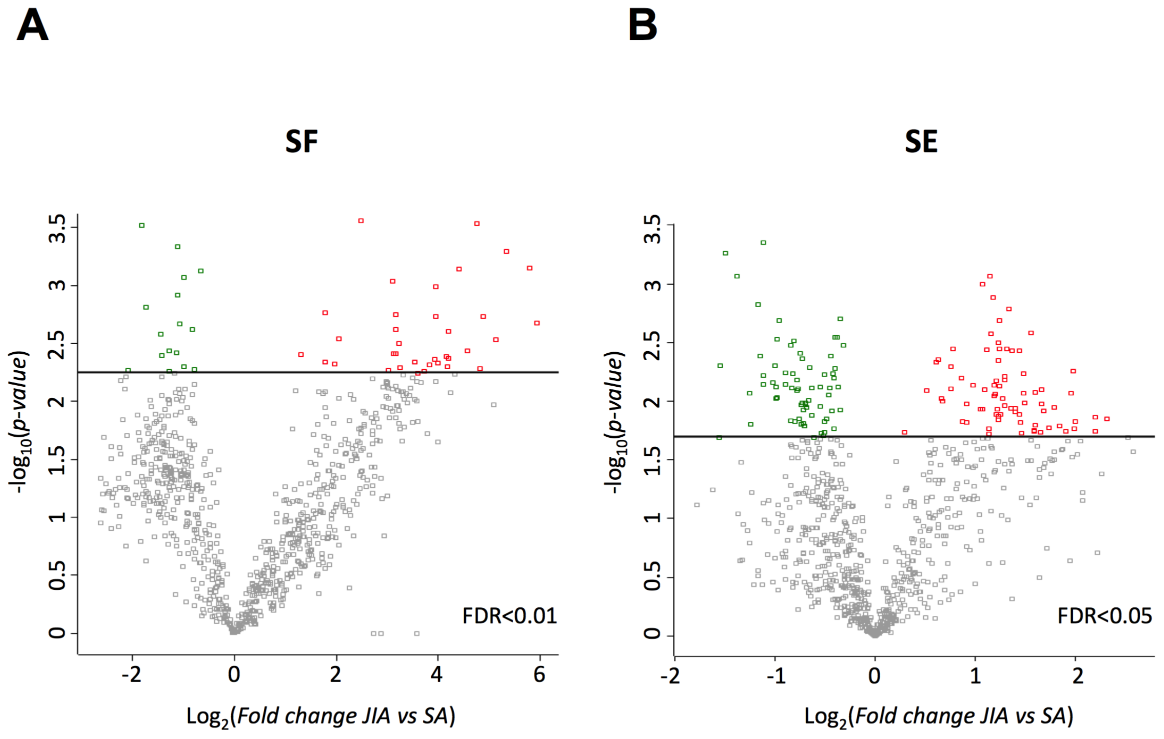

Fig. S2: Volcano plot of differentially miRNAs expressed in juvenile rheumatoid arthritis in comparison with septic arthritis identified by WTA.

miRNA with a FDR corrected *p-value* < 0.01 or 0.05 are depicted for synovial fluid (**A**) or serum (**B**) sample, respectively. Data points represent individual miRNA plotted as -log (FDR) versus $\log_2$fold change. Red or green points in the plot represent the differentially expressed miRNA with statistical significance with up- or down- deregulated in inflammatory arthritis comparing with septic disease.

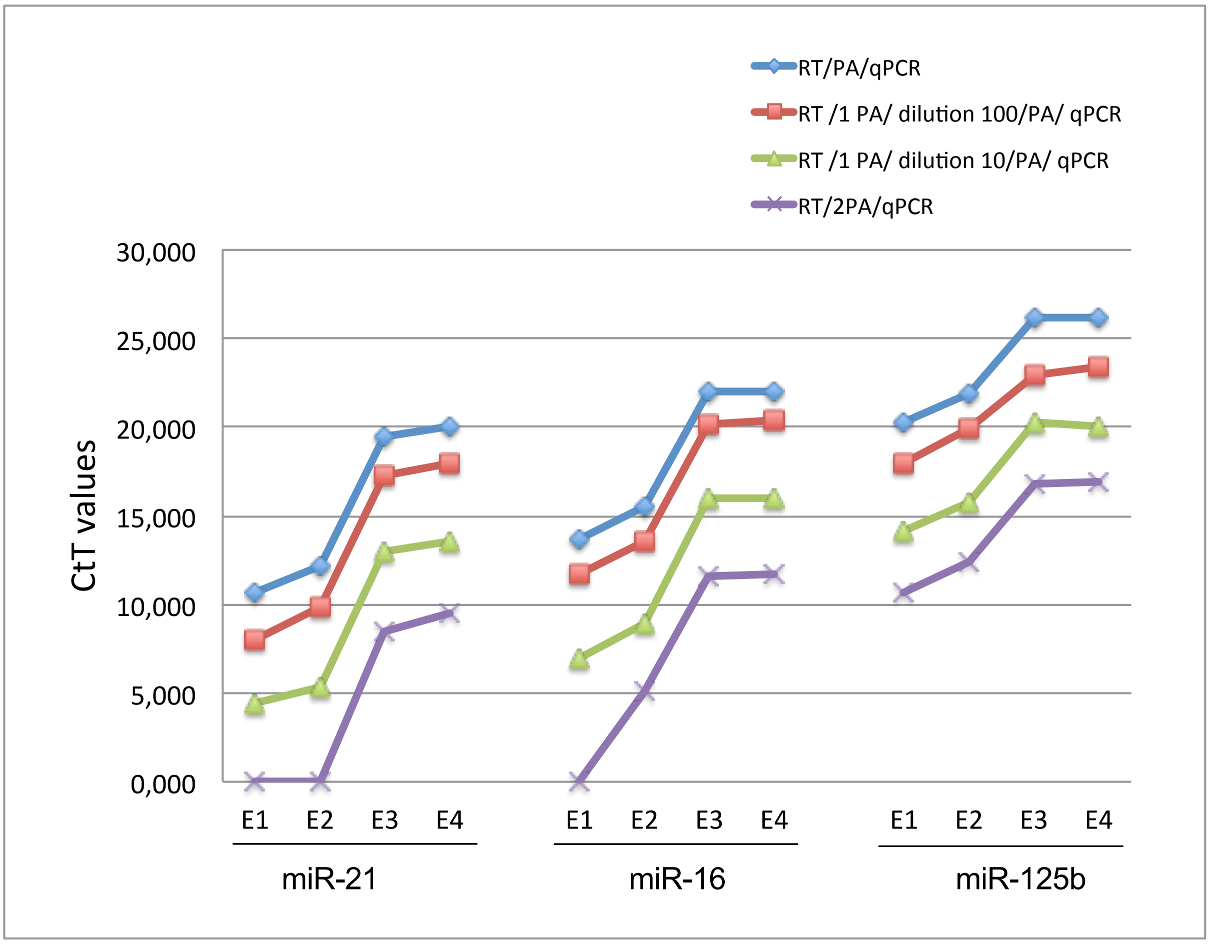

Fig. S3: Double preamplification validation

Total RNAs, including small RNAs, were extracted from 100 µl of 4 independent biological fluids (E1, E2, E3 and E4) using a miRNeasy Serum/Plasma kit with a Qiacube (QIAGEN) according to the manufacturer's instruction. For each sample eluent (from the sample isolation procedure) 2µL was used to perform reverse transcription, following preamplification. In blue, CT values obtained after preamplification and qPCR (classical protocol from life technology) are represented. In red or green, first pre-amplification products diluted 100 or 10 times, before preamplifcation and qPCR are showed. Finally, first preamplification products that were used directly for second preamplification before qPCR are represented in violet.

miRNA name	Log_2(FC)	miRNA name	Log_2(FC)	miRNA name	Log_2(FC)	miRNA name	Log_2(FC)
hsa-miR-6841-3p	-2,24	hsa-miR-150-5p	5,95	hsa-miR-5196-5p	3,39	hsa-miR-3648	2,92
hsa-miR-2909	-2,19	hsa-miR-7150	5,79	hsa-miR-6127	3,37	hsa-miR-4530	2,92
hsa-miR-8063	-2,14	hsa-miR-4417	5,34	hsa-miR-149-3p	3,34	hsa-miR-7109-5p	2,84
hsa-miR-6764-5p	-2,12	HK_SNORA66	5,12	hsa-miR-6778-5p	3,33	hsa-miR-7846-3p	2,79
hsa-miR-6734-3p	-2,09	hsa-miR-146a-5p	5,08	hsa-miR-6865-5p	3,32	hsa-miR-6894-5p	2,78
hsa-miR-3690	-1,90	hsa-miR-6716-5p	4,89	hsa-miR-671-5p	3,31	hsa-miR-6756-5p	2,74
hsa-miR-15b-3p	-1,82	hsa-miR-4800-5p	4,83	hsa-miR-6852-3p	3,31	hsa-miR-7106-5p	2,72
hsa-miR-6782-3p	-1,73	hsa-miR-6782-5p	4,76	hsa-miR-2861	3,27	hsa-miR-663b	2,72
hsa-miR-6748-3p	-1,73	hsa-miR-342-5p	4,57	hsa-miR-6877-5p	3,25	hsa-miR-3131	2,71
let.7a.2-3p	-1,65	hsa-miR-3687	4,42	hsa-miR-6821-3p	3,24	hsa-miR-6132	2,70
HK_RPS12	-1,63	hsa-miR-339-3p	4,32	hsa-miR-2392	3,24	hsa-miR-4487	2,57
hsa-miR-3926	-1,54	hsa-miR-648	4,24	hsa-miR-1908-5p	3,23	hsa-miR-1914-3p	2,55
hsa-miR-2116-3p	-1,45	hsa-miR-4646-5p	4,21	hsa-miR-4309	3,22	hsa-miR-4253	2,55
hsa-miR-4269	-1,43	hsa-miR-4419a	4,21	hsa-miR-1343-5p	3,22	hsa-miR-1275	2,54
hsa-miR-1244	-1,43	hsa-miR-4419b	4,19	hsa-miR-6769a-5p	3,21	hsa-miR-4461	2,49
hsa-miR-5589-5p	-1,42	hsa-miR-6794-5p	4,16	hsa-miR-766-5p	3,18	hsa-miR-4728-5p	2,49
hsa-miR-3619-3p	-1,37	hsa-miR-4667-5p	4,01	hsa-miR-6789-5p	3,17	hsa-miR-920	2,48
HK_PPIA	-1,30	hsa-miR-155-5p	4,00	hsa-miR-4484	3,17	hsa-miR-4688	2,45
hsa-miR-7156-3p	-1,29	hsa-miR-4442	3,96	hsa-miR-6875-5p	3,16	hsa-miR-501-5p	2,34
hsa-miR-18a-3p	-1,28	hsa-miR-1291	3,95	hsa-miR-1237-5p	3,16	hsa-miR-6870-3p	2,28
hsa-miR-6801-5p	-1,27	hsa-miR-4291	3,95	hsa-miR-33b-5p	3,16	hsa-miR-1307-5p	2,26
hsa-miR-1322	-1,27	hsa-miR-5703	3,92	hsa-miR-4257	3,13	hsa-miR-1307-3p	2,05
hsa-miR-885-5p	-1,18	hsa-miR-4463	3,83	hsa-miR-3141	3,12	hsa-miR-3180	2,05
hsa-miR-1976	-1,16	hsa-miR-6088	3,78	hsa-miR-1225-5p	3,11	hsa-miR-320a	1,96
hsa-miR-6729-3p	-1,15	hsa-miR-765	3,72	hsa-miR-6741-5p	3,10	hsa-miR-3140-3p	1,95
hsa-miR-1270	-1,14	hsa-miR-6797-5p	3,66	hsa-miR-4270	3,06	hsa-miR-3180-3p	1,79
hsa-miR-6731-3p	-1,12	hsa-miR-3912-3p	3,60	hsa-miR-7845-5p	3,04	hsa-miR-6780b-5p	1,78
hsa-miR-761	-1,11	hsa-miR-711	3,60	hsa-miR-6880-5p	3,03	hsa-miR-320d	1,78
hsa-miR-128.1-5p	-1,09	hsa-miR-4644	3,59	hsa-miR-6133	3,03	hsa-miR-4429	1,78
hsa-miR-146a-3p	-1,07	hsa-miR-8085	3,59	hsa-miR-23a-5p	3,02	hsa-miR-6723-5p	1,78
hsa-miR-1200	-1,07	hsa-miR-4478	3,55	hsa-miR-4505	3,00	hsa-miR-320c	1,74
hsa-miR-1306-5p	-1,00	hsa-miR-6870-5p	3,54	hsa-miR-625-5p	3,00	hsa-miR-320b	1,66
hsa-miR-1183	-0,99	hsa-miR-4665-5p	3,50	hsa-miR-6085	2,99	hsa-miR-5008-5p	1,31
hsa-miR-1202	-0,83	hsa-miR-4430	3,50	hsa-miR-4534	2,99	hsa-miR-1299	1,20
hsa-miR-130b-5p	-0,67	hsa-miR-6776-5p	3,46	hsa-miR-4784	2,97		
		hsa-miR-4481	3,44	hsa-miR-7108-5p	2,97		

Table S1: Liste of 141 miRNAs deregulated in synovial fluid

Fold change (FC) of miRNA expression in JIA versus SA.

	Synovial Fluid<br>AUC 95% CI
**miR-155-5p**	**0.997 95% CI [0.917-1]**
**miR-150-5p**	**0.959 95% CI [0.76-0.997]**
**miR-146a-5p**	**0.948 95% CI [0.734-0.995]**
**miR-6764-5p**	**0.926 95% CI [0.689-0.991]**
**miR-342-5p**	**0.915 95% CI [0.669-0.989]**
**miR-339-3p**	0.634 95% CI [0.5-0.862]
**miR-223-5p**	0.593 95% CI [0.5-0.835]
**miR-6841-5p**	0.563 95% CI [0.5-0.81]
**miR-648**	0.518 95% CI [0.5-0.732]
**miR-4519**	ND

Table S2: ROC curve analysis of miRNAs in synovial fluid and serum.

Abbreviations: AUC, area under the curve; CI, confidence interval; ROC, receiver operating characteristic; ND: not determined.
